# Profile of general hospitals in the Unified Health System

**DOI:** 10.11606/s1518-8787.2020054001982

**Published:** 2020-08-10

**Authors:** Laura de Almeida Botega, Mônica Viegas Andrade, Gilvan Ramalho Guedes

**Affiliations:** I Universidade Federal de Minas Gerais Faculdade de Ciências Econômicas Programa de Pós-graduação em Economia Belo HorizonteMG Brasil Universidade Federal de Minas Gerais. Faculdade de Ciências Econômicas. Programa de Pós-graduação em Economia. Belo Horizonte, MG, Brasil; II Universidade Federal de Minas Gerais Centro de Desenvolvimento e Planejamento Regional Belo HorizonteMG Brasil Universidade Federal de Minas Gerais. Centro de Desenvolvimento e Planejamento Regional. Belo Horizonte, MG, Brasil

**Keywords:** Hospitals, General, organization & administration, indicators, Health care, classification, Hospital Administration, Brazilian Unified Health System

## Abstract

**OBJECTIVE:**

To characterize the organization of Brazilian general hospitals that provide services to the Unified Health System using indicators that describe the main dimensions of hospital care.

**METHODS:**

A 2015 cross-sectional observational study, comprising the range of general hospitals that serve the Unified Health System. We constructed the hospital indicators from two national administrative databases: the National Registry of Health Facilities and the Hospital Information System of the Unified Health System. The indicators include the main dimensions associated with hospital care: public-private mix, production, production factors, performance, quality, case-mix and geographic coverage. Latent class analysis of indicators with bootstrapping was used to identify hospital profiles.

**RESULTS:**

We identified three profiles, with hospital size being the variable with the highest degree of belonging. Small hospitals show low occupancy rates (21.36%) and high participation of hospitalizations that could have been solved with outpatient care, besides attending only medium complexity cases. They receive few non-residents, indicating that they are mainly dedicated to the local population. Medium-sized hospitals are more similar to small-sized ones: about 100% of the visits are of medium complexity, low occupancy rate (45.81%), high rate of hospitalizations for primary care sensitive conditions (17.10%) and relative importance in the healthcare provision of non-residents (26%). Large hospitals provide high complexity care, have an average occupancy rate of 64.73% and show greater geographical coverage.

**CONCLUSIONS:**

The indicators point to three hospital profiles, characterized mainly by the production scale. Small hospitals show low performance, suggesting the need to reorganize hospital care provision, especially at the municipal level. The set of proposed indicators includes the main dimensions of hospital care, providing a tool that can help to plan and continuously monitor the hospital network of the Unified Health System.

## INTRODUCTION

In Brazil, as in almost all countries, hospital expense is an important component in total health expenditures. From 2010 to 2014, it represented an average of 36%, close to that observed for countries of the Organization for Economic Cooperation and Development (OECD), 38%^1,2^. The hospital sector’s high participation in total health expenditure is mainly due to the nature of the services provided. Unlike most production units, hospitals are characterized as multiproductive units of diagnostic and treatment services that require specialized infrastructure and intensive use of technologies and human resources. Additionally, the introduction of new technologies in hospital care is dynamic, continuously generating new equipment, medications and processes. These new technologies, besides being more expensive, are generally not substitutive, resulting in increased spending.

Hospital expenses may also be associated with the nature of the provider and how the hospital is managed, the hiring and payment systems of the providers and the presence of demand induction^3^. The hospital management type determines the level of administrative autonomy, directly impacting input purchase decisions (inventory control systems), the ability to introduce care protocols and the existence of cost management and care organization systems^8^. Private hospitals generally have greater management autonomy, which ends up resulting in risk management systems, security and more computerized and integrated costs, besides presenting a greater chance of modernization^10^. Hiring and payment models define the incentive structure under which providers will perform care^7,9^. In systems with predominance of payment per procedure, for example, there are clearly incentives to increase production^6,8^. In Brazil, especially in the private sector, payment per procedure prevails, while public hospitals show a greater diversity of remuneration structures^7^. In the Unified Health System (SUS), there is direct hiring of physicians and professionals in hospitals, in which remuneration is by salary. There is also payment by Authorization of Hospital Admissions (AIH) and payment per procedures, as in high complexity and outpatient care^7^.

Finally, the presence of demand induction is one of the most important elements in explaining the growing health expenditures in almost all countries. It is mainly related to financial incentives and the preferences of providers who have decision-making power, especially hospital care^3^, and is further intensified with the dynamism of the market in creating new technologies^8^. For most health care, especially curative, the principle of consumer sovereignty is invalid, becoming the provider’s responsibility choose the health service to be consumed, which determines an opportunity for demand induction.

Another important element that impacts the hospital sector’s performance is the health system organization and consequently how health services are delivered. In the SUS, care organization is decentralized, which requires coordination of the federative entities in allocating hospital resources. To ensure hospital expenditure efficiency, the system organization should consider the presence of economies of scale and scope in provisioning these services^11,12^. This is a challenge for Brazil, whose geopolitical configuration is marked by small municipalities, which, besides low population scale, have reduced technical management capacity and scarcity of human resources and equipment^13^.

Given this context, hospital management in the SUS is complex. It combines very diverse institutional arrangements that include multiple interaction of public and private providers, besides being governed by different administrative levels that have great regional and socioeconomic heterogeneity. In the absence of hospital care planning, one way to analyze their performance and measure control over its funding is by monitoring indicators. Benchmarking analysis allows us to observe the sector’s performance based on best practices, and monitoring hospital indicators helps ensuring a more efficient use of resources. OECD countries periodically monitor hospital indicators^2^.

It is common to have different perspectives on performance and hospital efficiency analyses. These terms can be thought of differently according to economics, public health or operational research principles. The influence and interdependence in different contexts of hospital care, however, hinders to think about these concepts separately. In this study, performance refer to studies dedicated to constructing and analyzing hospital indicators, while efficiency refers to studies focused on data envelopment analysis (DEA).

For Brazil, some studies have already measured hospital indicators considering specific sets of hospitals^9,14^. According to these analyses, hospital performance varies mainly according to the different sizes and forms of governance. For the whole country, there is only one not very recent study about 2002, using data from the National Health Facility Survey (AMS)^9^. The lack of studies for the country as a whole is partly due to the absence of reliable data at the national level^19,20^. Data from the National Registry of Health Facilities (CNES) have only become broader and periodically updated in recent years, after regulation established by the Ministry of Health and the Brazilian Health Regulatory Agency (ANVISA)^19,20^. The existence of CNES and the possibility of crossing with the production information from the Hospital Information System of the Unified Health System (SIH/SUS) allows defining a set of indicators that can be systematically monitored by public administrators. This study explores the possibilities of constructing indicators for the range of Brazilian general hospitals from available official information. We propose indicators that include the main dimensions that should be considered when analyzing hospital care organization: production and production factors, public-private mix, performance, quality, case-mix and geographical coverage.

## METHODS

We used two official databases to construct the indicators: the CNES and the SIH/SUS^21,22^. The first is a mandatory national registration with information on installed capacity and human resources from all health facilities. As the hospital infrastructure is practically constant throughout the year, we selected the month of July as a time reference. SIH/SUS, in turn, has information on all hospitalizations financed by the SUS. Only type 1 AIH, called normal, were considered, because long-term hospitalizations (type 5 AIH) consist of very differentiated health care, such as psychiatric treatments. These databases were integrated using the CNES code as a single identifier. The indicators were built for 2015, period in which CNES data already show greater reliability^19^.

Of a total of 6,154 hospitals, 5,120 were general hospitals. To characterize hospitals that provide services to the SUS, we included only those that registered at least 50% of the beds allocated to the public system. Practically inoperative hospitals, with less than 50 hospitalizations over the year, were disregarded. Three other hospitals were removed for not showing register for physicians in their records. Thus, 1,616 hospitals were excluded from the analysis. In total, the study considered 3,504 general hospitals that treated SUS patients.

Initially, we defined seven dimensions to be analyzed, considering important aspects of the hospital process, as well as the available official information, as described in the Chart: (i) public-private mix; (ii) production; (iii) case-mix; (iv) production factors; (v) performance; (vi) quality; (vii) geographical coverage.

**Chart:** Hospital dimensions analyzed, indicators and calculation method.

Dimension	Indicator	Indicator calculation method^a^
Public-private mix	SUS beds (%)	(Total SUS beds/Total existing beds)*100
Production	Monthly volume of care provided	Total AIH/12
Case-mix	Medium complexity procedures (%)	(Total medium complexity AIH/Total AIH)*100
	High complexity procedures (%)	(Total high complexity AIH/Total AIH)*100
	Hospitalizations for primary care sensitive conditions – HPCSC (%)	(Total HPCSC/Total AIH)*100
Production factors^b^	Physicians/bed	Total standardized physicians/Total SUS beds
	Nurses/bed	Total standardized nurses/Total SUS beds
	Nursing assistants-technicians/bed	Total standardized nursing assistants and technicians/Total SUS beds
	Senior management professionals/bed	Total directors and managers/Total SUS beds
	Medium complexity technology employed/bed	Medium complexity equipment/ Total SUS beds
	High technology employed/bed	High complexity equipment/ Total SUS beds
	Standardized hospitalization expense (US$/hospitalization)	Total standardized AIH expenditure/Total AIH
Performance	Turnover index	Total hospital discharges and deaths/Total SUS beds
	Average length of stay (days)	Total days of stay/Total AIH
	Occupancy rate (%)	(Total days of stay/Total SUS beds)*100
Quality^c^	Standardized crude mortality rate (%)	(Total standardized deaths/Total AIH)*100
	Hospital transfers (%)	(Total transfers/Total AIH without death)*100
Geographic coverage	Average distance traveled by SUS patients (km)	Total distance traveled by patients/Total AIH
	Non-resident care	(Total non-residents AIH/Total AIH)*100

^a^ The variables were annualized to calculate the indicators.

^b^ Standardized staff according to the workload of 12 hours for physicians, 36 hours for nurses and 40 hours for nursing assistants and technicians. Senior management professionals were not standardized, as they follow a single workload. Medium complexity equipment was grouped according to NH5, NH6 and NH7 of the NIV_HIER variable. High complexity equipment corresponds to the NH8 category of the NIV_HIER variable. Hospitalization expenses were standardized according to the distribution of the seven most frequent diagnoses in Brazil in 2015, grouped according to ICD-10 chapters: 1) circulatory system; 2) injuries, poisonings and other external causes; 3) circulatory system; 4) pregnancy, childbirth and puerperium; 5) neoplasms; 6) digestive system; 7) infectious and parasitic. The remaining chapters were considered to form a single group.

^c^ Standardized mortality according to the distribution of hospital deaths in Brazil in 2015, according to the six most lethal causes in the ICD-10 chapters. We considered as standard: 1) infectious and parasitic; 2) circulatory system; 3) abnormal symptoms of clinical and laboratory tests; 4) neoplasms; 5) respiratory system; 6) nutritional and metabolic endocrine diseases. The other causes of death were considered to form a single group.

The public-private mix dimension informs how much the hospital is dedicated to caring for SUS patients; the higher the percentage of SUS beds, the greater their dependence on public system funding. This dimension directly impacts the production result variable, measured by the monthly volume of care provided to the SUS (number of AIH).

Differences in production composition (case-mix) may be the main source of variation in hospital costs, as they reflect the complexity and severity of treatments^8^. In the present study, case-mix was classified according to the complexity levels (medium and high) and the proportion of hospitalizations for primary care sensitive conditions (HPCSC), which correspond to least complex procedures that could have been solved in outpatient care^23^. The higher frequency of HPCSC, besides reflecting low resolution of primary care, also points to inadequate hospital management^23^. Hospitals with low occupancy rates, for example, tend to have a high proportion of this type of care^25^. Under Roemer’s law, a health care system can determine its own demand, even in saturated markets^25^. Although difficult to identify and measure, this type of hospitalization tends to occur more frequently when the hospital still has AIH quotas^24,25^.

The production factors dimension contemplates the technical efficiency of inputs, that is, the hospital’s ability to optimally combine the use of medical and non-medical professionals with that of equipment (technology). The financial resources dimension refers to the total value of hospital procedures remunerated for the payment of AIH, which is the expense information available in the national hospital scope.

Performance dimension indicators are those commonly used in the literature to analyze hospital performance^9^. The turnover index reflects the efficiency of available physical resources, being measured by the ratio of the number of visits that resulted in discharge (or death) by the number of hospital beds. Occupancy rate informs the degree of utilization of the available physical resources (beds). High occupancy rates in general are associated with better performance, but depend directly on the average length of stay, which, in turn, reflects the quality of care provided, the efficiency of clinical management or the case-mix of care provided^9^. Thus, one must analyze performance indicators together.

The quality dimension reflects the positive results in patient care. High percentages of transfers between hospitals point to a low resolution of the services provided. Hospital mortality rate may reflect the quality of medical care, but it is conditioned by the type of hospital case-mix.

The geographical coverage dimension indicates the degree of reference of a hospital. High influx of non-residents may indicate the low resolution of the services provided in the locations of origin. To calculate the indicator of the average distance traveled by SUS patients, we used information from the patient’s municipality of residence and the hospital’s localization, present in the SIH/SUS and CNES databases, and the shortest path to be traveled between municipalities considering multimodal transport^21,22,26^. Hospital indicators were constructed using Stata 14.0.

The variables of health professionals, expenditures and mortality were standardized to allow comparing these indicators between hospitals. Health professionals were standardized according to the workload. Expenditures and mortality rate were standardized according to the aggregate chapters of the 10th revision of the International Classification of Diseases (ICD-10).

To identify similar hospitals regarding indicators and, thus, define the most appropriate cut-out for analyzing the hospitals’ profile, we used the analysis of latent classes for clusters^27^. The latent classes model for clusters (LCM) assumes that a latent variable *x*, of a multinomial nature, exists, with each category representing a specific profile. The model uses *T* indicators *y*_*it*_ of the *i* sample elements and *R* covariates *z*_*ir*_^cov^, which condition the occurrence of *x*. Indicators *y*_*it*_ and covariates *z*_*ir*_^cov^ can take on any nature (continuous, nominal, ordinal or counting). The probabilistic structure of the MCL described below assumes the presence of covariates and the possibility of using direct effects. Direct effects model residual covariance between indicators and between indicators and covariates, even if conditioned in *x*. Under these premises, *y*_*i*_ density can be described as:

$$f(y_{i}|z_{i}{\;}^{cov})\;=\;\sum_{{}_{x=1}}^{K}\;P(x|z_{i}^{cov})\;\Pi_{h=1}^{H}\;f(y_{ih}|x,z_{i}^{cov})$$

Where $P(x|z_{i}^{cov})$ it corresponds to the probability of observing the latent variable (or each of its categories), which depends directly on the levels of the covariates. To include direct effects of indicators and between indicators and covariates, *T* indicators are divided into *H* groups. Thus, indicators belonging to the same *H* set remain correlated after the conditionality in *x* and *z*_*i*_^cov^ but those belonging to distinct *H* will be conditionally independent. Class-specific conditional distributions, $f\left(y_{ih}|x,z_{i}^{cov}\right)$, can take distinct exact shapes depending on the scale of the variables in each subset *h*.

Identifying each class probabilities of occurrence is given by:

$$P\left(x|z_{i}^{cov}\right)\;=\frac{exp\;\left(\eta_{x|z^{cov}}\right)}{\sum_{x'=1}^{K}\;exp\;\left(\eta_{x'|z^{cov}}\right)},\;where\;x=1,\;...,\;K,$$

where $\eta_{x|z^{cov}}\;=z^{cov}\;\gamma$, in which *γ* represents the effects of each covariate on the linear transformation of the probability of occurrence of each cluster. The model parameters are obtained by maximum likelihood. In the article, we considered the indicators of the proposed dimensions, and included as covariates: the hospital size (small: up to 50 beds, medium-sized: 51 to 150 beds and large: above 150 beds), the type of provider (municipal public, state public, federal public, private and philanthropic) and the purpose of teaching and research.

Finally, to define the ideal number of profiles, we used an estimated p-value per bootstrap, *pˆ*_*boot*_. The statistic –2*LL*(*difference*) estimated by bootstrap is suggested for models with continuous indicators^28^. Statistic $-2LL\left(difference\right)\;=\;-2*\left(LL_{H0}\;-\;LL_{H1}\right)$, is defined, i.e. a model with K profiles (under *H*_0_) is compared with a model with K+1 profiles (under *H*_1_). In this case, *pˆ*_*boot*_ is estimated as the proportion of bootstrap statistics larger than the –2*LL*(*difference*) in the original sample. Confidence interval for the *pˆ*_*boot*_ is generated by the standard error $s\left({\hat{p}}_{boot}\right)\;=\;\sqrt{{\hat{p}}_{boot}\;\left(1\;-\;{\hat{p}}_{boot}\right)/B}$, where *B* represents the number of replications. Values of *pˆ*_*boot*_ > 0.05 suggest a model with fewer profiles. All estimates were made using Latent Gold 5.1.

A series of latent cluster models was estimated with *k* = 1, ..., 10. To select the ideal number of clusters, both the Bayesian information criterion (BIC) and the classification error were observed. If the decrease in BIC is followed by a significant increase in the error classification after including an additional cluster, the most parsimonious model is chosen. Based on these two criteria, we opted for the three-cluster model with an initial classification error of 0.0079. Since all indicators are continuous (or counting) and the bootstrap test method was used, a three-cluster model was compared with another of four clusters. The likelihood ratio test was not significant for the 49 additional parameters, reinforcing the choice of the three-cluster solution.

After estimating the model, we analyzed the matrix of conditional bivariate residues and identified high residues (above 1) for the following pairs: 1) hospital size with SUS beds, occupancy rate, monthly volume of care provided, standardized crude mortality rate, senior management professionals/bed, non-resident care, HPCSC rate and standardized hospitalization expenditure; 2) type of provider with SUS beds, doctors/bed standardized; 3) teaching hospital providing healthcare to non-residents. This result indicates violation of the assumption of local independence. To ease this assumption and still ensure the interpretability of the model with minimal loss of parsimony, we included direct effects for all these pairs with high residues and a bootstrap test –2*LL*(*difference*) with 5,000 replications between this model and the original.

The three-cluster model with direct effects showed greater adherence to the data, with the classification error below 0.01 (0.0089). The bootstrap likelihood test comparing the three-cluster model with direct effects and the original three-cluster model was significant, suggesting that including parameters was important for replicating the patterns in the data. All direct effects showed significance at 1%, and conditional bivariate residues after the inclusion of direct effects were reduced to all pairs of indicator-indicators and indicators-covariates (below 1), to guarantee the traditional interpretability of the latent cluster model.

## RESULTS

The Figure shows the participation of each type of hospital, described by the covariates, in each cluster. The hospital size variable shows very distinct patterns for the three identified clusters. Most small hospitals (75.16%) presents the characteristics of cluster 1, called Class 1, while medium-sized hospitals are mostly (60.97%) represented by Class 2, and large hospitals (66.08%) by Class 3. Regarding the type of provider, the participation in the clusters is not as defined as that observed for the hospital size covariate. The type of provider of Class 1 is predominantly municipal public (49.48%), followed by philanthropic (28.27%) and private (16.56%). Class 2 comprises predominantly philanthropic (36.02%) providers, followed by municipal public (30.30%). Class 3 hospitals are predominantly philanthropic (41.12%), followed by state public (28.03%). Teaching and research hospitals are concentrated in Class 3, representing 26.29% of all hospitals in this class.

**Figure:**
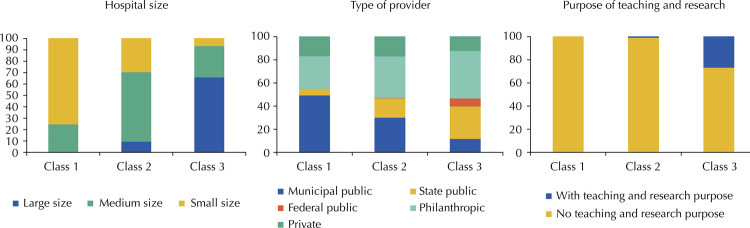
Degree of belonging (%) of the covariates of the model in the estimated clusters, Brazilian general hospitals, 2015.

Source: National Registry of Health Facilities and Hospital Information System of the Unified Health System, 2015^21,22^.

Table 1 shows the significance tests for the parameters of the indicators and estimated covariates. Wald and p-values tests show that all indicators are statistically significant at 1% among latent classes. The table also shows the coefficients of determination (R^2^) for each indicator of the latent class model. The R^2^measure indicates the degree to which the latent variable explains the variance of an indicator. The indicators analyzed that most explain clusters composition are the monthly volume of care provided (R^2^ = 0.4876), percentage of medium and high complexity procedures (R^2^ = 0.3560), expense per standardized hospitalization (R^2^ = 0.5103) and occupancy rate (R^2^ = 0.4892). Besides these indicators, non-resident care, crude mortality rates, HPCSC, average length of stay and nursing assistants and technicians per bed also stand out in defining the clusters, but with a lower degree of explanation, with R^2^ ranging from 0.22 to 0.25.

**Table 1:** Estimates of parameters on the form of linear projection, Brazilian general hospitals, 2015.

		Class 1	Class 2	Class 3	Wald	p	R^2^
	Number of SUS beds	-29.53	-16.41	45.94	137.20	0.000	0.4606
**Dimensions**	**Indicators**						
Public-private mix	SUS beds (%)	2.99	-0.26	-2.73	110.36	< 0.001	0.0766
Production	Monthly volume of care provided	-134.41	-35.32	169.73	222.21	< 0.001	0.4876
*Case-mix*	Medium complexity procedures (%)	3.92	3.86	-7.77	367.19	< 0.001	0.3560
	High complexity procedures (%)	-3.92	-3.86	7.77	367.19	< 0.001	0.3560
	Hospitalizations rates due to primary care sensitive conditions (HPCSC)	6.94	-1.14	-5.80	172.60	< 0.001	0.2370
Production factors	Standardized physicians/bed	-0.94	-0.15	1.09	323.69	< 0.001	0.1084
	Standardized nurses/bed	-0.15	-0.02	0.17	304.47	< 0.001	0.1516
	Standardized nursing assistants-technicians/bed	-0.49	-0.02	0.51	475.36	< 0.001	0.2225
	Senior management professionals/bed	-0.01	-0.01	0.02	10.04	0.0066	0.0040
	Medium complexity technology employed/bed	0.08	0.12	-0.20	100.75	< 0.001	0.0358
	High technology employed/bed	-0.76	-0.26	1.03	692.73	< 0.001	0.3891
	Standardized hospitalization expense (US$/hospitalization)*	-114.14	-54.93	169.06	229.19	< 0.001	0.5103
Performance	Average length of stay (days)	-1.62	-0.10	1.73	584.27	< 0.001	0.2430
	Turnover index	0.08	0.26	-0.34	39.03	< 0.001	0.0156
	Occupancy rate (%)	-17.94	3.66	14.28	459.75	< 0.001	0.4892
Quality	Standardized crude mortality rate (%)	-2.13	0.06	2.08	259.39	< 0.001	0.2684
	Hospital transfers (%)	-0.09	0.88	-0.80	18.31	< 0.001	0.0125
Geographic coverage	Non-resident care	-8.63	0.47	8.16	88.55	< 0.001	0.2519
	Average distance traveled by SUS patients (km)	-58.48	-15.52	74.00	166.54	< 0.001	0.0494

Source: National Registry of Health Facilities and Hospital Information System of the Unified Health System, 2015^21,22^.

* Average exchange rate for 2015 according to the historical series of the Central Bank of Brazil^29^.

Table 2 shows hospital indicators according to the estimated clusters . Most Brazilian general hospitals fall into Class 1 (59.77%), under municipal public management (49.48%), which performs a low average monthly volume of care (82.74 hospitalizations) and has a very low average occupancy rate (21.36%). This cluster encompasses hospitals that registered only medium complexity procedures and operate with a lower human capital intensity than the other clusters. The standardized crude hospital mortality rate (1.98%), below the other clusters, reflects the low degree of complexity of the care provided, as well as the resolution of the services provided. Moreover, the results show that many of the procedures performed in these hospitals should have been resolved in primary care (27.95%). These hospitals are practically used by the local population, as they serve only 11.01% of non-resident patients, whose average travel distance is 183.37 km (Table 2).

**Table 2:** Estimate of conditional averages of hospital indicators, Brazilian general hospitals, 2015.

		Class 1	Class 2	Class 3
	Cluster size (%)	59.77%	23.88%	16.34%
	Number of SUS beds	35.63	73.35	197.62
**Dimensions**	**Indicators**			
Public-private mix	SUS beds (%)	91.98	87.41	85.07
Production	Monthly volume of care provided	82.74	258.52	685.25
*Case-mix*	Medium complexity procedures (%)	100.00	99.94	88.31
	High complexity procedures (%)	0.01	0.06	11.69
	Hospitalizations rates due to primary care sensitive conditions (HPCSC)	27.95	17.10	10.59
Production factors	Standardized physicians/bed	0.50	1.30	2.88
	Standardized nurses/bed	0.13	0.26	0.46
	Standardized nursing assistants-technicians/bed	0.52	0.99	1.52
	Senior management professionals/bed	0.04	0.03	0.04
	Medium complexity technology employed/bed	0.48	0.53	0.20
	High technology employed/bed	0.00	0.50	1.79
	Standardized hospitalization expense (US$/hospitalization)^a^	117.53	196.75	473.88
Performance	Average length of stay (days)	3.11	4.63	6.45
	Turnover index	1.94	2.12	1.52
	Occupancy rate (%)	21.36	45.81	64.73
Quality	Standardized crude mortality rate (%)	1.98	4.52	6.89
	Hospital transfers (%)	3.59	4.55	2.88
Geographic coverage	Non-resident care	11.01	25.85	36.17
	Average distance traveled by SUS patients (km)	183.37	226.33	315.86

Source: National Registry of Health Facilities and Hospital Information System of the Unified Health System, 2015^21,22^.

^a^ Average exchange rate for 2015 according to the historical series of the Central Bank of Brazil^29^.

Class 3 hospitals, although representing 16.34% of the country’s hospitals, answer for a high volume of care: 685.25 hospitalizations per month, 88.31% of medium complexity and 11.69% of high complexity. And these are the hospitals that perform highly complex procedures. Hospitals in the cluster where large and teaching and research hospitals are more likely to occur, present more health human capital and high technology. The greater complexity of the care provided is also reflected in the average expenditure per hospitalization (473.88 US$/hospitalization), the average length of stay (6.45 days) and the standardized crude mortality rate (6.89%). Regarding senior management professionals, they are similar to the other classes. These hospitals operate with an average occupancy rate of 64.73%, close to the levels recommended by the National Agency of Health Insurance (ANS), from 75% to 85%^30^. They are also a reference in healthcare, receiving 36.17% of non-resident patients, who need to travel on average 315.86 km. It is worth noting that 10.59% of the hospitalizations performed in these hospitals should have occurred within the scope of primary care (Table 2).

Class 2 hospitals sit in an intermediate position, which is reflected in the indicators shown (Table 2). Regarding case-mix, they practically do not perform high complexity procedures, although they have equipment of this level. Of the hospitalizations performed, 17.10% are HPCSC, and the institutions operate with an occupancy rate (45.81%) below that recommended by the ANS. The important role in the care of non-resident patients (25.85%) is noteworthy.

## DISCUSSION

This study analyzes the profile of Brazilian general hospitals, presenting relevant results regarding their functioning and degree of importance in the public hospital network. The multidimensional analysis pointed to the hospitals’ different vocational profiles, which vary according to size, type of provider and purpose of teaching and research.

Small hospitals are predominantly public-municipal and, although they operate with occupancy rates well below that recommended by the ANS^30^, a result also observed in other studies^9,14^, they play an important role in caring for the local population. In addition, they perform high rates of hospitalizations that should have been attended in primary care (HPCSC). Larger hospitals are mostly public-state and philanthropic, have a high geographical coverage and perform a high monthly volume of visits, carrying out practically all procedures of high complexity of SUS patients. These hospitals had occupancy rates closer to those recommended by the ANS^30^, which was also verified in other studies on Brazil^9,14^and OECD countries in 2017^31^. Although many studies conducted with Brazilian teaching and research hospitals suggest that their peculiarities place them in a separate group^9,14,32^, the multidimensional analysis showed that their indicators are similar to those of large hospitals.

Despite the predominance of certain types of hospitals in each of the identified clusters, some hospitals are displaced, such as large hospitals in the smaller hospitals cluster. These hospitals seem to overlook their vocational roles, that is, small and public-municipal hospitals more focused on the problems of the surrounding population and larger hospitals as a reference in high complexity care, thus serving a greater proportion of non-residents. It would be important in a future research to identify and study these displaced hospitals.

Larger hospitals have installed capacity available to provide healthcare for additional patients, pointing to important opportunities for reorganizing the Brazilian hospital system. This reorganization, however, must consider not only hospital performance, but also the aspect of equity in access to hospital services. Small hospitals characterized by low performance may have their existence associated with the need to ensure access to hospital services, especially in remote areas. Most Brazilian municipalities lack population scale or financial capacity to offer more complex health care^33,34^. In this sense, municipal hospitals in small population cities generally play a limited role in the care network. The principle of decentralizing the SUS generates incentives for local managers to invest in installing small hospitals with low resolution, which operate more as a gateway to the system and are a reference for the local population. A coordination strategy is needed in defining and planning the location of hospitals. Some attempts to reorganize the supply have been made within the scope of the SUS, such as the regionalization and the intermunicipal consortia^35^. Neither of these two attempts, however, was sufficient to reorganize the supply and limit the incentives of local managers to maintain and install small municipal hospitals. Moreover, primary care is not yet an organizer of care in the SUS, where the logic of acute care still prevails, which is offered mainly in the hospital environment^34^.

In recent years, criticisms of hospital-centricity have marginalized hospital care regarding SUS strategic analyses^33^. The hospital system operates disconnected from the rest of the healthcare network^33,34^. From 2002 to 2015, there were no significant advances in the use of available resources in small hospitals^9^, which historically have been characterized by low occupancy rates, besides performing a high percentage of HPCSC^25^. In the current context of strong containment of public spending, improving hospital resources management would favor the continuity of service provision.

Some European Union countries sought to circumvent the issue of low performance of small hospitals through hospital reforms, whose strategies ranged from centralizing the provision of hospital services with closing hospitals (departments), through hospital mergers, to converting hospital beds into home beds^36,37^. Hospital reform in Portugal, for example, sought to circumvent the government’s budget crisis with management practices that aimed at promoting greater efficiency, access and quality for patients^36^; however, one limitation of this reform was that some hospitals merged, but their services continued to be performed in separate units, without significant efficiency gains^36^.

The main contribution of this study was to propose a set of indicators that allow analyzing the profile of hospitals according to different dimensions, which can be constructed from the available public information. These indicators were sufficient to characterize hospitals in different profiles. This study is unprecedented, mainly because it analyzes all general hospitals in Brazil, while prior studies concentrated on specific Brazilian hospitals groups^9,14^.

This study presents some limitations. First, given the complexity of medical care, indicators cannot incorporate all the particularities of the process, for example, physicians’ expertise, technological level of equipment and patients’ health conditions. Second, the study analyzes only hospitalizations financed by the SUS, although the types of providers are public, private and philanthropic. In addition, the figures presented correspond to the expenditure calculated by the AIH, disregarding complementary resources transferred to hospitals. Despite the possibility of record errors occurring in the CNES database, it is considered to be of good reliability^19^.

The results found in this study provide an overview of the Brazilian hospital sector, pointing to different operating profiles regarding hospital size, type of provider and purpose of teaching and research. The proposed set of indicators provides parameters that can contribute to the sector’s continuous monitoring and its construction can be automated by feeding the existing administrative bases. The analysis of these indicators does not exclude other types of approach, such as technical efficiency and scale analysis, which provide a comparative analysis of these hospitals’ performance.
