# Supplementary material for: Profile of general hospitals in the Unified Health System
Source: Rev Saude Publica. 2020 Aug 10;54:82. doi: 10.11606/s1518-8787.2020054001982 (PMC7416762; doi:10.11606/s1518-8787.2020054001982)
Supplement: Translation

# Translation: Perfil dos hospitais gerais do Sistema Único de Saúde

**Authors:** Laura de Almeida Botega, Mônica Viegas Andrade, Gilvan Ramalho Guedes

**Affiliations:** I Brasil Universidade Federal de Minas Gerais. Faculdade de Ciências Econômicas. Programa de Pós-graduação em Economia. Belo Horizonte, MG, Brasil; II Brasil Universidade Federal de Minas Gerais. Centro de Desenvolvimento e Planejamento Regional. Belo Horizonte, MG, Brasil

**Keywords:** Hospitais Gerais, organização & administração, indicadores, Assistência à Saúde, classificação, Administração Hospitalar, Sistema Único de Saúde

## Abstract

**OBJETIVO:**

Caracterizar a organização dos hospitais gerais brasileiros que prestam serviço ao Sistema Único de Saúde por meio de indicadores que descrevem as principais dimensões do cuidado hospitalar.

**MÉTODOS:**

Estudo observacional transversal para o ano de 2015, compreendendo o universo dos hospitais gerais que atendem o Sistema Único de Saúde. Os indicadores hospitalares foram construídos a partir de duas bases de dados administrativos nacionais: o Cadastro Nacional de Estabelecimento de Saúde e o Sistema de Informações Hospitalares do Sistema Único de Saúde. Os indicadores contemplam as principais dimensões associadas ao cuidado hospitalar: *mix* público-privado, produção, fatores de produção, desempenho, qualidade, *case-mix* e abrangência geográfica. A análise de classes latentes dos indicadores com implementação do *bootstrapping* foi utilizada para a identificação dos perfis hospitalares.

**RESULTADOS:**

Foram identificados três perfis, sendo porte hospitalar a variável com grau de pertencimento mais elevado. Os hospitais pequenos apresentam baixas taxas de ocupação (21,36%) e elevada participação de internações que poderiam ter sido solucionadas com cuidado ambulatorial, além de atenderem somente a média complexidade. Recebem poucos não residentes, indicando que estão dedicados praticamente à população local. Os hospitais de médio porte se assemelham mais aos de pequeno porte: cerca de 100% dos atendimentos são de média complexidade, baixa taxa de ocupação (45,81%), elevada taxa de internações por condições sensíveis à atenção primária (17,10%) e relativa importância no atendimento de não residentes (26%). Os hospitais grandes realizam os atendimentos de alta complexidade, têm taxa de ocupação média de 64,73% e apresentam maior abrangência geográfica.

**CONCLUSÕES:**

Os indicadores apontam três perfis de hospitais, caracterizados principalmente pela escala de produção. Os hospitais de pequeno porte apresentam baixa performance, sugerindo a necessidade de reorganização da oferta do cuidado hospitalar, principalmente no nível municipal. O conjunto dos indicadores propostos inclui as principais dimensões do cuidado hospitalar, fornecendo uma ferramenta que pode ser utilizada no planejamento e monitoramento contínuo da rede hospitalar do Sistema Único de Saúde.

## INTRODUÇÃO

No Brasil, assim como em quase todos os países, o gasto hospitalar é um componente importante nas despesas totais com saúde. No período de 2010 a 2014, representou em média 36%, próximo ao observado para os países da Organização para a Cooperação e Desenvolvimento Econômico (OCDE), 38%^1,2^. A elevada participação do setor hospitalar no gasto total com saúde se deve principalmente à natureza dos serviços prestados. Diferentemente da maior parte das unidades produtivas, os hospitais são caracterizados como unidades multiprodutoras de serviços de diagnóstico e tratamento que exigem uma infraestrutura especializada e uso intensivo de tecnologias e recursos humanos. Além disso, a introdução de novas tecnologias no cuidado hospitalar é dinâmica, verificando-se a geração contínua de novos equipamentos, medicamentos e processos. Essas novas tecnologias, além de mais dispendiosas, em geral não são substitutivas, determinando elevação dos gastos.

Os gastos hospitalares podem também estar associados à natureza do prestador e à forma de gestão do hospital, aos sistemas de contratação e de pagamento dos prestadores e à presença de indução de demanda^3^. O tipo de gestão hospitalar determina o nível de autonomia administrativa, impactando diretamente nas decisões de compra de insumos (sistemas de controle de estoque), capacidade de introdução de protocolos de cuidado e existência de sistemas de gerenciamento de custos e organização do cuidado^8^. Hospitais privados apresentam em geral maior autonomia de gestão, o que acaba resultando na presença de sistemas de gerenciamento de risco, segurança e custos mais informatizados e integrados, além de apresentarem maior chance de modernização^10^. As formas de contratação e de pagamento definem a estrutura de incentivos sob as quais os prestadores irão realizar o cuidado^7,9^. Em sistemas com predominância do pagamento por procedimento, por exemplo, há claramente incentivos à ampliação da produção^6,8^. No Brasil, principalmente no setor privado, predomina o pagamento por procedimento, enquanto nos hospitais públicos há maior diversidade de estruturas de remuneração^7^. No Sistema Único de Saúde (SUS), existe nos hospitais a contratação direta de médicos e profissionais. Neste caso, a remuneração é por salário. Há também o pagamento por meio de autorização de internações hospitalares (AIH) e o pagamento por procedimentos, como na alta complexidade e atenção ambulatorial^7^.

Por fim, a presença de indução de demanda é um dos elementos mais importantes na explicação dos gastos crescentes com saúde em quase todos os países. Ela está relacionada principalmente aos incentivos financeiros e às preferências dos prestadores que detêm o poder de decisão, sobretudo, do cuidado hospitalar^3^, sendo ainda intensificada com o dinamismo do mercado na criação de novas tecnologias^8^. Para a maior parte dos cuidados com a saúde, especialmente curativos, o princípio da soberania do consumidor não é válido, passando a ser de responsabilidade do prestador a escolha do serviço de saúde a ser consumido, o que determina uma oportunidade para a prática da indução de demanda.

Outro elemento importante que impacta a performance do setor hospitalar é a organização do sistema de saúde e consequentemente a forma de entrega dos serviços de saúde. No SUS, a organização do cuidado é realizada de forma descentralizada, o que exige uma coordenação dos entes federativos na alocação dos recursos hospitalares. Para garantir a eficiência do gasto hospitalar, a organização do sistema deveria considerar a presença de economias de escala e escopo no provimento desses serviços^11,12^. Esse é um desafio para o Brasil, cuja configuração geopolítica é marcada por municípios de pequeno porte, que, além de baixa escala populacional, apresentam reduzida capacidade técnica de gestão e escassez de recursos humanos e equipamentos^13^.

Nesse contexto, a gestão hospitalar no SUS é complexa. Ela combina arranjos institucionais muito diversificados que incluem uma interação múltipla de prestadores públicos e privados, assim como é regida por diferentes níveis administrativos e que guardam grande heterogeneidade regional e socioeconômica. Na ausência de um planejamento do cuidado hospitalar, uma forma de analisar a sua performance e aferir controle sobre seu financiamento é pelo acompanhamento de indicadores. A análise de *benchmarking* possibilita observar o desempenho do setor com base nas melhores práticas, sendo o monitoramento de indicadores hospitalares uma forma de garantir o uso mais eficiente dos recursos. Nos países da OCDE há um acompanhamento periódico de indicadores hospitalares^2^.

É comum haver perspectivas distintas sobre análises de performance, desempenho e eficiência hospitalar. Esses termos podem ser pensados de forma diferente segundo os princípios da economia, saúde pública ou pesquisa operacional. A influência e interdependência em diferentes contextos do cuidado hospitalar, no entanto, torna difícil pensar nesses conceitos separadamente. Neste trabalho, os termos desempenho e performance referem-se aos estudos que se dedicam à construção e análise de indicadores hospitalares, enquanto o conceito de eficiência refere-se aos estudos direcionados à análise de envoltória de dados (DEA).

Para o Brasil, alguns trabalhos já mensuraram indicadores hospitalares considerando conjuntos específicos de hospitais^9,14^. Segundo esses estudos, a performance hospitalar varia principalmente entre os diferentes portes e formas de governança. Para o país como um todo, há apenas um estudo não muito recente sobre o ano de 2002, utilizando os dados da pesquisa de assistência médico-sanitária (AMS)^9^. A escassez de estudos para o país como um todo se deve em parte à ausência de dados confiáveis no nível nacional^19,20^. Os dados do Cadastro Nacional de Estabelecimentos de Saúde (CNES) só passaram a ser mais ampla e periodicamente alimentados nos últimos anos, após regulamentação estabelecida pelo Ministério da Saúde e pela Agência Nacional de Vigilância Sanitária (Anvisa)^19,20^. A existência do CNES e a possibilidade de cruzamento com as informações de produção do Sistema de Informações Hospitalares do Sistema Único de Saúde (SIH/SUS) abrem uma janela de oportunidade para a definição de um conjunto de indicadores que podem ser acompanhados de forma sistemática pelos gestores. Esse trabalho explora as possibilidades de construção de indicadores para o universo dos hospitais gerais brasileiros a partir das informações oficiais disponíveis. São propostos indicadores que contemplam as principais dimensões que devem ser consideradas na análise da organização do cuidado hospitalar: produção e fatores de produção, *mix* público-privado, desempenho, qualidade, *case-mix* e abrangência geográfica.

## MÉTODOS

Dois bancos de dados oficiais foram utilizados para a construção dos indicadores: o CNES e o SIH/SUS^21,22^. O primeiro é um registro nacional de preenchimento obrigatório com informações de capacidade instalada e recursos humanos de todos os estabelecimentos de saúde. Como a infraestrutura hospitalar é praticamente constante ao longo do ano, selecionamos o mês de julho como referência temporal. Já o SIH/SUS possui informações de todas as internações financiadas pelo SUS. Foram consideradas apenas as AIH tipo 1, denominadas normais, pois as internações de longa duração (AIH tipo 5) consistem em cuidados de saúde muito diferenciados, como tratamentos psiquiátricos. Essas bases foram integradas utilizando-se o código CNES como um identificador único. Os indicadores foram construídos para o ano de 2015, período em que os dados do CNES já apresentam maior confiabilidade^19^.

De um total de 6.154 hospitais, 5.120 eram hospitais gerais. Para caracterizar os hospitais que prestam serviços ao SUS, foram incluídos somente os que registraram pelo menos 50% dos leitos alocados para o sistema público. Os hospitais praticamente inoperantes, com menos de 50 internações ao longo do ano, foram desconsiderados. Além disso, três hospitais não foram analisados, pois não havia registros para médicos em seus cadastros. Dessa forma, 1.616 hospitais foram excluídos da análise. No total, o estudo considerou 3.504 hospitais gerais que atenderam pacientes do SUS.

Inicialmente foram definidas sete dimensões a serem analisadas, considerando aspectos importantes do processo hospitalar, assim como a disponibilidade de informações oficiais, conforme descrito no Quadro: (i) *mix* público-privado; (ii) produção; (iii) *case-mix*; (iv) fatores de produção; (v) desempenho; (vi) qualidade; (vii) abrangência geográfica.

**Quadro:** Dimensões hospitalares analisadas, indicadores e método de cálculo.

Dimensão	Indicador	Método de cálculo do indicador ^a^
*Mix* público-privado	Leitos SUS (%)	(Total de leitos SUS/Total de leitos existentes)*100
Produção	Volume de atendimentos prestados no mês	Total de AIH/12
*Case-mix*	Procedimentos de média complexidade (%)	(Total de AIH de média complexidade/Total de AIH)*100
	Procedimentos de alta complexidade (%)	(Total de AIH de alta complexidade/Total de AIH)*100
	Internações por condições sensíveis à atenção primária - ICSAP (%)	(Total de ICSAP/Total de AIH)*100
Fatores de produção ^b^	Médicos/leito	Total de médicos padronizados/Total de leitos SUS
	Enfermeiros/leito	Total de enfermeiros padronizados/Total de leitos SUS
	Auxiliares-técnicos enfermagem/leito	Total de auxiliares e técnicos de enfermagem padronizados/Total de leitos SUS
	Profissionais da alta gestão/leito	Total de diretores e gerentes/ Total de leitos SUS
	Tecnologia de média complexidade empregada/leito	Equipamentos de média complexidade/ Total de leitos SUS
	Alta tecnologia empregada/leito	Equipamentos de alta complexidade/ Total de leitos SUS
	Gasto por internação padronizado (US$/internação)	Gasto total das AIH padronizados/Total de AIH
Desempenho	Índice de rotatividade	Total de altas hospitalares e óbitos/Total de leitos SUS
	Tempo médio de permanência (dias)	Total de dias de permanência/Total de AIH
	Taxa de ocupação (%)	(Total de dias de permanência/Total de leitos SUS)*100
Qualidade ^c^	Taxa bruta de mortalidade padronizada (%)	(Total de óbitos padronizados/Total de AIH)*100
	Transferências hospitalares (%)	(Total de transferências/Total de AIH sem óbito)*100
Abrangência geográfica	Distância média percorrida pelos pacientes do SUS (km)	Total da distância percorrida pelos pacientes/Total de AIH
	Atendimento não-residentes	(Total de AIH de não-residentes/Total de AIH)*100

^a^ As variáveis foram anualizadas para o cálculo dos indicadores.

^b^ Pessoal padronizado segundo a carga horária de 12 horas para médicos, 36 horas para enfermeiros e 40 horas para auxiliares e técnicos de enfermagem. Os profissionais da alta gestão não foram padronizados, pois seguem uma carga horária única. Equipamentos de média complexidade foram agrupados segundo NH5, NH6 e NH7 da variável NIV_HIER. Equipamentos de alta complexidade correspondem a categoria NH8 da variável NIV_HIER. Gasto por internação foi padronizado segundo a distribuição dos sete diagnósticos mais freqüentes no Brasil em 2015, agrupados segundo os capítulos da CID-10: 1) aparelho circulatório; 2) lesões, envenenamentos e outras causas externas; 3) aparelho circulatório; 4) gravidez, parto e puerpério; 5) neoplasias; 6) aparelho digestivo; 7) infecciosas e parasitárias. Os demais capítulos foram considerados como compondo um único grupo.

^c^ Mortalidade padronizada segundo a distribuição dos óbitos hospitalares do Brasil em 2015, segundo as seis causas mais letais dos capítulos da CID-10, foi considerada como padrão: 1) infecciosas e parasitárias; 2) aparelho circulatório; 3) sintomas anormais de exames clínicos e laboratoriais; 4) neoplasias; 5) aparelho respiratório; 6) doenças endócrinas nutricionais e metabólicas. As demais causas de morte foram consideradas como compondo um único grupo.

A dimensão *mix* público-privado informa o quanto o hospital está dedicado ao atendimento de pacientes do SUS; quanto maior o percentual de leitos SUS, maior sua dependência ao financiamento do sistema público. Essa dimensão impacta diretamente a variável de resultado de produção, mensurada pelo volume mensal de atendimentos prestados ao SUS (número de AIH).

Diferenças na composição da produção (*case-mix*) podem ser a principal fonte de variação nos custos hospitalares, pois refletem a complexidade e severidade dos tratamentos^8^. No presente trabalho, o *case-mix* foi classificado segundo os níveis de complexidade (médio e alto) e a proporção de internações por condições sensíveis à atenção primária (ICSAP), que correspondem aos procedimentos de menor complexidade que poderiam ter sido resolvidos no âmbito da atenção primária^23^. A maior frequência de ICSAP, além de refletir baixa resolutividade da atenção primária, também aponta para inadequação da gestão hospitalar^23^. Hospitais com baixa taxa de ocupação, por exemplo, tendem a apresentar proporção elevada desse tipo de atendimento^25^. Segundo a lei de Roemer, um sistema de saúde tem a habilidade de determinar sua própria demanda, mesmo em mercados saturados^25^. Embora de difícil identificação e mensuração, sabe-se que esse tipo de internação tende a ocorrer com maior frequência quando o hospital ainda tem quotas de AIH^24,25^.

A dimensão dos fatores de produção contempla a eficiência técnica dos insumos, isto é, a capacidade do hospital de combinar otimamente o uso de profissionais médicos e não médicos com o de equipamentos (tecnologia). A dimensão dos recursos financeiros se refere ao valor total dos procedimentos hospitalares remunerados pelo pagamento das AIH, que se constitui na informação de despesa disponível no âmbito hospitalar nacionalmente.

Os indicadores na dimensão desempenho constituem aqueles comumente utilizados pela literatura para analisar a performance hospitalar^9^. O índice de rotatividade reflete a eficiência dos recursos físicos disponíveis e é medido pela razão do número de atendimentos que resultaram em alta (ou em óbito) pelo número de leitos do hospital. A taxa de ocupação informa o grau de utilização dos recursos físicos (leitos) disponíveis. Altas taxas de ocupação em geral estão associadas a uma melhor performance, mas dependem diretamente do tempo médio de permanência, que, por sua vez, reflete a qualidade do atendimento prestado, a eficiência da gestão clínica ou o *case-mix* do atendimento prestado^9^. Dessa forma, é importante analisar os indicadores de desempenho em conjunto.

A dimensão da qualidade reflete os resultados positivos no atendimento aos pacientes. Altos percentuais de transferências entre hospitais apontam para uma baixa resolutividade dos serviços prestados. A taxa de mortalidade hospitalar pode refletir a qualidade do cuidado médico, mas está condicionada ao tipo de *case-mix* do hospital.

A dimensão abrangência geográfica indica o grau de referência de um hospital. O elevado influxo de não residentes pode indicar a baixa resolutividade dos serviços prestados nas localidades de origem. Para calcular o indicador da distância média percorrida pelos pacientes do SUS, foi utilizada a informação do município de residência do paciente e do município de localização do hospital, presentes nas bases de dados do SIH/SUS e do CNES, e o menor caminho a ser percorrido entre os municípios considerando transporte multimodal^21,22,26^. Os indicadores hospitalares foram construídos utilizando-se o *software* Stata 14.0.

As variáveis de profissionais da saúde, gastos e mortalidade foram padronizadas para possibilitar a comparação desses indicadores entre os hospitais. Os profissionais de saúde foram padronizados de acordo com a carga horária trabalhada. Os gastos e a taxa de mortalidade foram padronizados segundo os capítulos agregados da 10ª revisão da Classificação Internacional de Doenças (CID-10).

Para identificar hospitais similares em termos dos indicadores e, como consequência, definir o recorte mais apropriado para a análise do perfil dos hospitais, foi utilizada neste estudo a análise de classes latentes para *clusters*^27^. O modelo de classes latentes para *clusters* (MCL) assume que existe uma variável latente, *x*, de natureza multinomial, com cada categoria representando um perfil específico. O modelo utiliza *T* indicadores *y*_*it*_ dos *i* elementos amostrais e *R* covariáveis *z*_*ir*_^cov^, as quais condicionam a ocorrência de *x*. Os indicadores *y*_*it*_ e as covariáveis *z*_*ir*_^cov^ podem assumir qualquer natureza (contínua, nominal, ordinal ou de contagem). A estrutura probabilística do MCL descrita a seguir assume a presença de covariáveis e a possibilidade de uso de efeitos diretos. Os efeitos diretos modelam a covariância residual entre indicadores e entre indicadores e covariáveis, mesmo condicionados em *x*. Sob essas premissas, a densidade de *y*_*i*_ pode ser descrita como:

$$f(y_{i}|z_{i}{\;}^{cov})\;=\;\sum_{{}_{x=1}}^{K}\;P(x|z_{i}^{cov})\;\Pi_{h=1}^{H}\;f(y_{ih}|x,z_{i}^{cov})$$

em que $P(x|z_{i}^{cov})$ corresponde à probabilidade de observar a variável latente (ou cada uma de suas categorias), a qual depende diretamente dos níveis das covariáveis. Para incluir efeitos diretos de indicadores e entre indicadores e covariáveis, agrupam-se os *T* indicadores em *H* grupos. Assim, os indicadores que pertencem ao mesmo conjunto *H* continuam correlacionados após a condicionalidade em *x* e *z*_*i*_^cov^, mas os que pertencem a *H* distintos serão condicionalmente independentes. As distribuições condicionais, específicas por classe, $f\left(y_{ih}|x,z_{i}^{cov}\right)$, podem ter formas exatas distintas dependendo da escala das variáveis em cada subconjunto *h*.

A identificação das probabilidades de ocorrência de cada classe é dada por:

$$P\left(x|z_{i}^{cov}\right)\;=\frac{exp\;\left(\eta_{x|z^{cov}}\right)}{\sum_{x'=1}^{K}\;exp\;\left(\eta_{x'|z^{cov}}\right)},\;onde\;x=1,\;...,\;K,$$

em que $\eta_{x|z^{cov}}\;=z^{cov}\;\gamma$, onde *γ* representa os efeitos de cada covariável sobre a transformação linear da probabilidade de ocorrência de cada *cluster*. Os parâmetros do modelo são obtidos por máxima verossimilhança. No artigo foram considerados os indicadores das dimensões propostas, e como covariáveis foram incluídos o porte hospitalar (pequeno: até 50 leitos, médio: 51 a 150 leitos e grande: acima de 150 leitos), o tipo de prestador (público municipal, público estadual, público federal, privado e filantrópico) e a finalidade de ensino e pesquisa.

Por fim, para definir o número ideal de perfis, utilizou-se um p-valor estimado por *bootstrap, pˆ*_*boot*_. A estatística –2*LL*(*difference*) estimada por *bootstrap* é sugerida para modelos com indicadores contínuos^28^. Define-se a estatística $-2LL\left(difference\right)\;=\;-2*\left(LL_{H0}\;-\;LL_{H1}\right)$, ou seja, compara-se um modelo com K perfis (sob *H*_0_) com um modelo com K+1 perfis (sob *H*_1_). Nesse caso, o *pˆ*_*boot*_ é estimado como a proporção das estatísticas de *bootstrap* maiores que a –2*LL*(*difference*) da amostra original. Para gerar o intervalo de confiança para o *pˆ*_*boot*_, utiliza-se o erro-padrão $s\left({\hat{p}}_{boot}\right)\;=\;\sqrt{{\hat{p}}_{boot}\;\left(1\;-\;{\hat{p}}_{boot}\right)/B}$, em que *B* representa o número de replicações. Valores de *pˆ*_*boot*_ > 0.05 sugerem um modelo com menos perfis. Todas as estimativas foram realizadas utilizando-se o *software* Latent Gold 5.1.

Foi estimada uma série de modelos de *cluster* latentes, com *k* = 1, ..., 10. Com o objetivo de selecionar o número ideal de *clusters*, foram observados tanto o critério de informação bayesiano (BIC) como o erro de classificação. Se a diminuição no BIC for seguida de um aumento significativo na classificação do erro após a inclusão de um *cluster* adicional, opta-se pelo modelo mais parcimonioso. Com base nesses dois critérios, optou-se pelo modelo de três *clusters* com um erro de classificação inicial de 0,0079. Uma vez que todos os indicadores são contínuos (ou de contagem) e utilizou-se o método de teste de *bootstrap*, comparou-se um modelo de três clusters com outro de quatro *clusters*. O teste da razão de verossimilhança não foi significativo para os 49 parâmetros adicionais, reforçando a escolha da solução de três *clusters*.

Após a estimação do modelo, analisou-se a matriz de resíduos bivariados condicionais e foram identificados resíduos elevados (acima de 1) para os seguintes pares: 1) porte hospitalar com leitos SUS, taxa de ocupação, volume de atendimentos prestados no mês, taxa bruta de mortalidade padronizada, profissionais de alta gestão/leito, atendimento de não residentes, taxa de ICSAP e gasto por internação padronizado; 2) tipo de prestador com leitos SUS, médicos/leito padronizado; 3) hospital de ensino com atendimento de não residentes. Esse resultado é indicativo de violação do pressuposto de independência local. Para relaxar esse pressuposto e garantir ainda a interpretabilidade do modelo com mínima perda de parcimônia, foram incluídos efeitos diretos para todos esses pares com resíduos elevados e feito um teste –2*LL*(*difference*) por *bootstrap* com 5.000 replicações entre esse modelo e o modelo original.

O modelo de três *clusters* com efeitos diretos apresentou maior aderência aos dados, com o erro de classificação abaixo de 0,01 (0,0089). O teste de verossimilhança do *bootstrap* comparando o modelo de três *clusters* com efeitos diretos e o modelo de três *clusters* original foi significativo, sugerindo que a inclusão de parâmetros foi importante para replicar os padrões nos dados. Todos os efeitos diretos apresentaram significância a 1%, e os resíduos bivariados condicionais após a inclusão dos efeitos diretos foram reduzidos para todos os pares de indicadores-indicadores e indicadores-covariáveis (abaixo de 1), de modo a garantir a interpretabilidade tradicional do modelo de *cluster* latente.

## RESULTADOS

A Figura apresenta a participação de cada tipo de hospital, descrito pelas covariáveis, em cada um dos *clusters*. A variável porte mostra padrões bem distintos para os três *clusters* identificados. A maior parte dos hospitais de pequeno porte (75,16%) apresenta as características do *cluster* 1, denominado Classe 1, enquanto os hospitais de médio porte estão em sua maioria (60,97%) representados pela Classe 2, e os hospitais de grande porte (66,08%) pela Classe 3. Em relação ao tipo de prestador, a participação nos *clusters* não é tão definida como a observada para a covariável porte. O tipo de prestador da Classe 1 é predominantemente público municipal (49,48%), seguido dos filantrópicos (28,27%) e privados (16,56%). Na Classe 2 estão predominantemente os filantrópicos (36,02%), seguidos dos públicos municipais (30,30%). Os hospitais da Classe 3 são predominantemente filantrópicos (41,12%), seguidos dos públicos estaduais (28,03%). Os hospitais de ensino e pesquisa estão concentrados na Classe 3, representando 26,29% do total de hospitais dessa classe.

**Figura:** Grau de pertencimento (%) das covariáveis do modelo nos *clusters* estimados, hospitais gerais brasileiros, 2015.

Fonte: Cadastro Nacional de Estabelecimentos de Saúde e Sistema de Informações Hospitalares do Sistema Único de Saúde, 2015^21,22^.

A Tabela 1 apresenta os testes de significância para os parâmetros dos indicadores e covariáveis estimados. Os testes de Wald e dos p-valores mostram que todos os indicadores são estatisticamente significativos a 1% entre as classes latentes. Na tabela também são apresentados os coeficientes de determinação (R^2^) de cada indicador do modelo de classes latentes. A medida do R^2^indica o grau no qual a variância de um indicador é explicada pela variável latente. Os indicadores analisados que mais explicam a composição dos *clusters* são volume de atendimentos prestados no mês (R^2^ = 0,4876), percentual de procedimentos de média e alta complexidade (R^2^ = 0,3560), gasto por internação padronizado (R^2^ = 0,5103) e taxa de ocupação (R^2^ = 0,4892). Além desses indicadores, atendimentos de não residentes, taxas brutas de mortalidade, ICSAP, tempo médio de permanência e auxiliares e técnicos de enfermagem por leito também se destacam na definição dos *clusters*, mas com um grau de explicação menor, com R^2^ variando de 0,22 a 0,25.

**Tabela 1:** Estimativas dos parâmetros sobre a forma de projeção linear, hospitais gerais brasileiros, 2015.

		Classe 1	Classe 2	Classe 3	Wald	p	R^2^
	Quantidade de Leitos SUS	-29,53	-16,41	45,94	137,20	0,000	0,4606
**Dimensões**	**Indicadores**						
*Mix* público-privado	Leitos SUS (%)	2,99	-0,26	-2,73	110,36	< 0,001	0,0766
Produção	Volume de atendimentos prestados no mês	-134,41	-35,32	169,73	222,21	< 0,001	0,4876
*Case-mix*	Procedimentos de média complexidade (%)	3,92	3,86	-7,77	367,19	< 0,001	0,3560
	Procedimentos de alta complexidade (%)	-3,92	-3,86	7,77	367,19	< 0,001	0,3560
	Taxas de Internações por condições sensíveis à atenção primária (ICSAP)	6,94	-1,14	-5,80	172,60	< 0,001	0,2370
Fatores de produção	Médicos/leito padronizado	-0,94	-0,15	1,09	323,69	< 0,001	0,1084
	Enfermeiros/leito padronizado	-0,15	-0,02	0,17	304,47	< 0,001	0,1516
	Auxiliares-técnicos enfermagem/leito padronizado	-0,49	-0,02	0,51	475,36	< 0,001	0,2225
	Profissionais da alta gestão/leito	-0,01	-0,01	0,02	10,04	0,0066	0,0040
	Tecnologia de média complexidade empregada/leito	0,08	0,12	-0,20	100,75	< 0,001	0,0358
	Alta tecnologia empregada/leito	-0,76	-0,26	1,03	692,73	< 0,001	0,3891
	Gasto por internação padronizado (US$/internação)*	-114,14	-54,93	169,06	229,19	< 0,001	0,5103
Desempenho	Tempo médio de permanência (dias)	-1,62	-0,10	1,73	584,27	< 0,001	0,2430
	Índice de rotatividade	0,08	0,26	-0,34	39,03	< 0,001	0,0156
	Taxa de ocupação (%)	-17,94	3,66	14,28	459,75	< 0,001	0,4892
Qualidade	Taxa bruta de mortalidade padronizada (%)	-2,13	0,06	2,08	259,39	< 0,001	0,2684
	Transferências hospitalares (%)	-0,09	0,88	-0,80	18,31	< 0,001	0,0125
Abrangência geográfica	Atendimento não-residentes	-8,63	0,47	8,16	88,55	< 0,001	0,2519
	Distância média percorrida pelos pacientes do SUS (km)	-58,48	-15,52	74,00	166,54	< 0,001	0,0494

Fonte: Cadastro Nacional de Estabelecimentos de Saúde e Sistema de Informações Hospitalares do Sistema Único de Saúde, 2015^21,22^.

* Câmbio médio de 2015 segundo as séries históricas do Banco Central do Brasil^29^.

A Tabela 2 apresenta os indicadores hospitalares segundo os *clusters* estimados. A maior parte dos hospitais gerais brasileiros está na Classe 1 (59,77%), sob a gestão pública municipal (49.48%), a qual realiza reduzido volume médio mensal de atendimentos (82,74 internações) e apresenta taxa média de ocupação muito baixa (21,36%). Nesse *cluster*, estão hospitais que registraram apenas procedimentos de média complexidade e operam com uma intensidade de capital humano inferior aos demais *clusters*. A taxa bruta de mortalidade hospitalar padronizada (1,98%), abaixo dos demais *clusters*, reflete o baixo grau de complexidade dos atendimentos prestados, assim como a resolutividade dos serviços prestados. Além disso, os resultados mostram que muitos dos procedimentos realizados nesses hospitais deveriam ter sido resolvidos no âmbito da atenção primária (27,95%). Esses hospitais são praticamente utilizados pela população local, pois atendem apenas 11,01% de pacientes não residentes, cuja distância média de deslocamento é de 183,37 km (Tabela 2).

**Tabela 2:** Estimativa de médias condicionais dos indicadores hospitalares, hospitais gerais brasileiros, 2015.

		Classe 1	Classe 2	Classe 3
	Tamanho do Cluster (%)	59,77%	23,88%	16,34%
	Quantidade de Leitos SUS	35,63	73,35	197,62
**Dimensões**	**Indicadores**			
*Mix* público-privado	Leitos SUS (%)	91,98	87,41	85,07
Produção	Volume de atendimentos prestados no mês	82,74	258,52	685,25
*Case-mix*	Procedimentos de média complexidade (%)	100,00	99,94	88,31
	Procedimentos de alta complexidade (%)	0,01	0,06	11,69
	Taxas de internações por condições sensíveis à atenção Primária (ICSAP)	27,95	17,10	10,59
Fatores de produção	Médicos/leito padronizado	0,50	1,30	2,88
	Enfermeiros/leito padronizado	0,13	0,26	0,46
	Auxiliares- técnicos enfermagem/leito padronizado	0,52	0,99	1,52
	Profissionais da alta gestão/leito	0,04	0,03	0,04
	Tecnologia de média complexidade empregada/leito	0,48	0,53	0,20
	Alta tecnologia empregada/leito	0,00	0,50	1,79
	Gasto por internação padronizado (US$/internação)^a^	117,53	196,75	473,88
Desempenho	Tempo médio de permanência (dias)	3,11	4,63	6,45
	Índice de rotatividade	1,94	2,12	1,52
	Taxa de ocupação (%)	21,36	45,81	64,73
Qualidade	Taxa bruta de mortalidade padronizada (%)	1,98	4,52	6,89
	Transferências hospitalares (%)	3,59	4,55	2,88
Abrangência geográfica	Atendimento não-residentes	11,01	25,85	36,17
	Distância média percorrida pelos pacientes do SUS (km)	183,37	226,33	315,86

Fonte: Cadastro Nacional de Estabelecimentos de Saúde e Sistema de Informações Hospitalares do Sistema Único de Saúde, 2015^21,22^.

^a^ Câmbio médio de 2015 segundo as séries históricas do Banco Central do Brasil^29^.

Os hospitais da Classe 3, embora representem 16,34% dos hospitais do país, são responsáveis por elevado volume de atendimentos: 685,25 internações por mês, sendo 88,31% de média complexidade e 11,69% de alta complexidade. E é nesses hospitais que os procedimentos de alta complexidade são realizados. Os hospitais do *cluster* em que os hospitais de grande porte e de ensino e pesquisa têm maior probabilidade de ocorrência são mais intensivos em capital humano da área da saúde e em alta tecnologia. A maior complexidade dos atendimentos prestados é também refletida no gasto médio por internação (473,88 US$/internação), no tempo médio de permanência (6,45 dias) e na taxa bruta de mortalidade padronizada (6,89%). Já em termos de profissionais da alta gestão, não se diferenciam muito. Esses hospitais operam com uma taxa média de ocupação, de 64,73%, situando-se próximos dos níveis preconizados pela Agência Nacional de Saúde Suplementar (ANS), de 75% a 85%^30^. São também referência no atendimento, recebendo 36,17% de pacientes não residentes, que precisam se deslocar em média 315,86 km. Vale observar que 10,59% das internações realizadas nesses hospitais deveriam ter ocorrido no âmbito da atenção primária (Tabela 2).

Os hospitais da Classe 2 situam-se em uma posição intermediária, o que se reflete nos indicadores apresentados (Tabela 2). Em relação ao *case-mix*, praticamente não realizam procedimentos de alta complexidade, ainda que possuam equipamentos desse nível. Das internações realizadas, 17,10% são ICSAP, e as instituições operam com taxa de ocupação (45,81%) abaixo do recomendado pela ANS. Chama a atenção o importante papel no atendimento de pacientes não residentes (25,85%).

## DISCUSSÃO

Este estudo faz uma análise do perfil dos hospitais gerais brasileiros, apresentando resultados relevantes quanto a seu funcionamento e grau de importância na rede hospitalar pública. A análise multidimensional apontou para diferentes perfis vocacionais dos hospitais, que variam segundo porte, tipo de prestador e finalidade de ensino e pesquisa.

Os hospitais pequenos são predominantemente público-municipais e, embora operem com taxas de ocupação muito abaixo do recomendado pela ANS^30^, resultado que também foi observado em outros estudos^9,14^, possuem um importante papel no atendimento à população local. Além disso, realizam altas taxas de internações que deveriam ter sido atendidas na atenção primária (ICSAP). Os hospitais de maior porte são em sua maioria público-estaduais e filantrópicos, possuem elevada abrangência geográfica e realizam um elevado volume de atendimentos mensal, sendo neles realizados praticamente todos os procedimentos de alta complexidade dos pacientes do SUS. Esses hospitais apresentaram taxas de ocupação mais próximas do recomendado pela ANS^30^, o que também foi verificado em outros trabalhos sobre o Brasil^9,14^e países da OCDE em 2017^31^. Embora muitos estudos tenham sido feitos com os hospitais de ensino e pesquisa brasileiros, sugerindo que suas peculiaridades os colocam em um grupo separado^9,14,32^, a análise multidimensional mostrou que seus indicadores se assemelham aos dos hospitais de grande porte.

Apesar do predomínio de determinados tipos de hospitais em cada um dos *clusters* identificados, alguns hospitais se encontram deslocados, como hospitais grandes no *cluster* de hospitais menores. Esses hospitais parecem, portanto, não desempenhar seus papéis vocacionais, isto é, hospitais pequenos e público-municipais mais voltados aos problemas da população circunjacente e hospitais maiores como referência em atendimentos de alta complexidade, atendendo, portanto, uma maior proporção de não residentes. Seria importante em um futuro trabalho identificar e estudar esses hospitais deslocados.

Os hospitais maiores possuem capacidade instalada disponível para atender pacientes adicionais, apontando para oportunidades importantes de reorganização do sistema hospitalar brasileiro. Essa reorganização, no entanto, precisa considerar não apenas o desempenho hospitalar, mas também o aspecto de equidade no acesso aos serviços hospitalares. Os hospitais de pequeno porte caracterizados por apresentar baixo desempenho podem ter sua existência associada à necessidade de garantir acesso aos serviços hospitalares, principalmente em áreas remotas. A maioria dos municípios brasileiros não possui escala populacional ou capacidade financeira para ofertar cuidados de saúde mais complexos^33,34^. Nesse sentido, hospitais municipais em cidades de pequeno porte populacional apresentam em geral um limitado papel na rede de cuidados. O princípio da descentralização do SUS gera incentivos para que os gestores locais invistam na instalação de pequenos hospitais de baixa resolutividade, que operam mais como porta de entrada para o sistema e são referência para a população local. É necessária uma estratégia de coordenação na definição e planejamento da localização dos hospitais. Algumas tentativas para reorganizar a oferta têm sido realizadas no âmbito do SUS, como a regionalização e a formação de consórcios intermunicipais^35^. Nenhuma dessas duas tentativas, no entanto, foi suficiente para reorganizar a oferta e limitar os incentivos dos gestores locais à manutenção e instalação de hospitais de pequeno porte municipais. Além disso, a atenção primária ainda não é ordenadora do cuidado no SUS, onde ainda impera a lógica do cuidado agudo, o qual é ofertado sobretudo no ambiente hospitalar^34^.

Nos últimos anos, as críticas em relação ao hospitalocentrismo fizeram com que a atenção hospitalar fosse marginalizada em termos de análises estratégicas do SUS^33^. O sistema hospitalar funciona de forma desarticulada do restante da rede assistencial^33,34^. De 2002 a 2015, não houve avanços significativos na utilização dos recursos disponíveis nos hospitais de pequeno porte^9^, que historicamente têm se caracterizado por apresentar baixas taxas de ocupação, além de realizar elevado percentual de ICSAP^25^. No contexto atual de forte contenção dos gastos públicos, uma melhoria na gestão dos recursos hospitalares favoreceria a continuidade do provimento dos serviços.

Alguns países da União Europeia buscaram contornar a questão do baixo desempenho dos hospitais de pequeno porte por meio de reformas hospitalares, cujas estratégias variaram de centralização da provisão dos serviços hospitalares com fechamento de hospitais (departamentos), passando por fusões hospitalares, até conversão de leitos hospitalares em leitos domiciliares^36,37^. A reforma hospitalar em Portugal, por exemplo, buscou contornar a crise orçamentária do governo com práticas de gestão que tinham como objetivo promover maior eficiência, acesso e qualidade para os pacientes^36^; entretanto, uma limitação dessa reforma foi que alguns hospitais se fundiram, mas seus serviços continuaram a ser realizados em unidades separadas, sem ganhos significativos de eficiência^36^.

A principal contribuição deste trabalho foi propor um conjunto de indicadores que permitem analisar o perfil dos hospitais segundo diferentes dimensões e que podem ser construídos a partir das informações públicas disponíveis. Esses indicadores foram suficientes para caracterizar os hospitais em diferentes perfis. Este estudo é inédito, sobretudo por analisar a totalidade dos hospitais gerais no Brasil, uma vez que os estudos que o precedem se concentraram em grupos específicos de hospitais brasileiros^9,14^.

Este trabalho apresenta algumas limitações. Em primeiro lugar, dada a complexidade do cuidado médico, os indicadores não conseguem incorporar todas as particularidades do processo, por exemplo, expertise dos médicos, nível tecnológico dos equipamentos e condições de saúde dos pacientes. Em segundo lugar, o estudo analisa apenas as internações financiadas pelo SUS, embora os tipos de prestadores sejam públicos, privados e filantrópicos. Além disso, os valores apresentados correspondem ao gasto apurado pelas AIH, não considerando os recursos complementares repassados aos hospitais. Apesar da possibilidade de ocorrência de erros de registros na base de dados do CNES, essa base é considerada de boa confiabilidade^19^.

Os resultados encontrados neste trabalho fornecem um panorama do setor hospitalar brasileiro, apontando para distintos perfis de funcionamento em termos de porte hospitalar, tipo de prestador e finalidade de ensino e pesquisa. O conjunto de indicadores proposto fornece parâmetros que podem contribuir para o monitoramento contínuo do setor e sua construção pode ser automatizada pela alimentação das bases administrativas já existentes. A análise desses indicadores não exclui outros tipos de abordagem, como análises de eficiência técnica e de escala, que fornecem uma análise comparativa do desempenho desses hospitais.
